# A human cancer-associated truncation of MBD4 causes dominant negative impairment of DNA repair in colon cancer cells

**DOI:** 10.1038/sj.bjc.6603592

**Published:** 2007-02-06

**Authors:** S A Bader, M Walker, D J Harrison

**Affiliations:** 1 Department of Pathology, Edinburgh Cancer Research Centre, University of Edinburgh, Crewe Road, Edinburgh EH4 2XR, UK

**Keywords:** MBD4, colorectal cancer, genomic instability, Big Blue, mutation frequency

## Abstract

MBD4 binds to methylated DNA and acts as a thymine DNA glycosylase in base excision repair. Deficiency of MBD4 in mice enhances mutation at CpG sites and alters apoptosis in response to DNA damage, but does not increase tumorigenesis in mismatch repair-deficient mice. However, in humans, frameshift mutation of MBD4, rather than deletion, is what occurs in up to 43% of microsatellite unstable colon cancers. There is no murine equivalent of this mutation. We now show that recombinant truncated MBD4 (MBD4^tru^) inhibits glycosylase activities of normal MBD4 or Uracil DNA glycosylase in cell-free assays as a dominant negative effect. Furthermore, overexpression of *MBD4*^*tru*^ in Big Blue (*lacI*)-transfected, MSI human colorectal carcinoma cells doubled mutation frequency, indicating that the modest dominant negative effect on DNA repair can occur in living cells in short-term experiments. Intriguingly, the whole mutation spectrum was increased, not only at CpG sites, suggesting that truncated MBD4 has a more widespread effect on genomic stability. This demonstration of a dominant negative effect may be of significance in tumour progression and acquisition of drug resistance.

MBD4 is a methyl-CpG-binding DNA glycosylase involved especially in the repair of mismatches arising from deamination of methyl-C in mammalian cells. MBD4 has been shown *in vitro* to excise mismatched thymine (T) bases from oligo templates (Hendrich *et al*, 1999; Petronzelli *et al*, 2000a, 2000b), and mouse knockout models have found that in the absence of Mbd4, mutation frequency (MF) *in vivo* increases, mainly at methyl-CpG sites (Millar *et al*, 2002; Wong *et al*, 2002). MBD4 can also bind to MLH1 and Fas-associated death domain (FADD) proteins (Bellacosa *et al*, 1999; Screaton *et al*, 2003), and the small intestine of Mbd4^−/−^ mice shows reduced apoptosis in response to a variety of DNA-damaging agents (Cortellino *et al*, 2003; Sansom *et al*, 2003; Screaton *et al*, 2003). Absence of Mbd4 in mice also increases tumorigenicity in the tumour-susceptible APC^min^ background (Millar *et al*, 2002; Wong *et al*, 2002). The tumorigenic effect may be due to an increase in MF, decreased apoptosis or a combination of both. However, absence of Mbd4 in an MSH2-null background does not increase tumorigenicity above that seen owing to the MMR-deficiency (Sansom *et al*, 2004).

In humans, a naturally occurring frameshift mutation of a polynucleotide tract in *MBD4* has been found in microsatellite unstable (MSI) colon and other cancers (Bader *et al*, 1999; Riccio *et al*, 1999; Menoyo *et al*, 2001; Yamada *et al*, 2002) that leads to a premature stop in translation of the mRNA. The mutation occurs in 20–43% of cases (mixed tissue samples), whereas microdissected samples (enriched for tumour cells) of 89% sporadic MSI colon tumours had truncated MBD4 mutations in at least one focus tested (Bader *et al*, 2000). Crucial in this study in human cells, for comparison with prior studies in mice, the equivalent mutation of *Mbd4* is not present in MSI mouse models as the murine gene does not contain a coding polyA microsatellite. The *Mbd4*-null phenotype modeled in mice is an experimental condition only, as in humans mutation or loss of *MBD4* only occurs in the presence of the frame-shifted allele. Although mutation in *MBD4* is thought to occur in MSI tumours as a consequence of the microsatellite instability caused by mismatch repair (MMR) protein inactivation, we have postulated that *MBD4* mutation may still affect tumour progression in humans. Bi-allelic inactivation of *MBD4* (by frameshift mutation of both alleles, or of one allele with chromosomal loss of the second) has been seen in a small number of cases or samples (Bellacosa *et al*, 1999; Bader *et al*, 2000). However, by far the most common condition appears to be frameshift of one allele in the presence of a remaining normal allele, and there has been no documented evidence of the null genotype as studied in the mouse models. A recombinant N-terminal portion of MBD4 containing amino acids (aa) 1–165 including the MBD has been shown to be still able to bind to methyl-CpG oligos including also those with T·G mismatches (Hendrich *et al*, 1999). It is therefore possible that the naturally-occurring *MBD4* mutation exerts a dominant negative effect, where the truncated protein is not only defective in glycosylase activity, but also can inhibit normal MBD4 function via competitive binding of mismatch sites. In this study, we describe how truncated MBD4 can inhibit normal glycosylase activity in a cell-free system, and increase mutation frequency across a wide spectrum of mutation changes in living colon cancer cells even on a background of pre-existing MSI.

## MATERIALS AND METHODS

### Recombinant proteins, *in vitro* transcription/translation and Western immunoblots

For MBD4^tru^, random primed cDNA was made from HCT116 cells and PCR amplified with forward primer 4NcoMet and MBD4.12ABam. PCR product was double digested with *NcoI* and *BamHI* and cloned into *Nco*I+*BamH*I digested bacterial expression vector pET6H. Recombinant proteins were expressed in *Escherichia coli* BLR (*λ*DE3)/pLysS and purified over nickel-agarose columns (Qiagen, West Sussex, UK) followed by Fractogel EMD SO_3_^2−^ 650(M) columns (Merck) as described (Free *et al*, 2001). MBD4 proteins were also made by *in vitro* transcription/translation using linearised MBD4 cDNA-containing pET6H plasmids in T_N_T T_7-_coupled reticulocyte lysate (Promega, Hampshire, UK) in the presence of [^35^S] Methionine.

Nuclear extracts from cell lines were made using the Proteo Extract subcellular proteome extraction kit (Calbiochem, Nottinghamshire, UK) according to the manufacturer's instructions.

For Western immunoblotting, proteins were subjected to SDS–PAGE on 10% BisTris NuPage gels (Invitrogen, Paisley, UK) in MOPS buffer, blotted onto Hybond P membrane (Amersham, Buckinghamshire, UK) and probed with antibodies using standard protocols. Antibodies used were monoclonal anti-His tag (Novagen, Nottinghamshire, UK), polyclonal anti-MBD4 (H-300, raised against amino acids 281–580 and capable of binding to truncated MBD4 with normal sequence up to residue 313, Santa Cruz) and monoclonal anti-lamin B (Calbiochem).

### Glycosylase assays

Assays were performed essentially as described before (Hendrich *et al*, 1999), with modification of the piperidine cleavage assay involving the inclusion not only of the active wt recombinant glycosylase (MBD4 or UDG) but also a range of 0–100 ng MBD4^tru^ as competitor in a constant volume. Briefly, 10 ng recombinant wtMBD4 or 0.2 U uracil DNA glycosylase (New England Biolabs, Hertfordshire, UK) protein was incubated with annealed oligonucleotides (the reverse strand of each pair having been end-labelled with [^33^P] ATP) with or without recombinant MBD4^tru^ in a constant total volume of 40 *μ*l. Primer combinations (Hendrich and Bird, 1998; Hendrich *et al*, 1999) used were BH:BHrev, BH:BHrevU, MM2:MM3revU.

### DNA for transfection

A portion of 2 *μ*g of *λ*LIZ (Big Blue shuttle vector, Stratagene, Cambridgeshire, UK) DNA was methylated at 37°C for 30 min *in vitro* using excess *Sss*I *methylase* according to the manufacturer's protocol (New England Biolabs), and then concatenated for 1 h at room temperature using DNA ligase (New England Biolabs) before transfection. MBD4^tru^ cDNA was made by cloning the PCR product from HCT116 cDNA (primers MBD4.1a and MBD4.14) into pGEMT (Promega) and subsequently it was transferred into pcDNA3 (Invitrogen). cDNA insert was sequenced by cycle sequencing to confirm identity and presence of mutant A_9_ polynucleotide stretch as well as lack of any other introduced mutations.

### Cell lines and culture, including transfection

All cell lines except HCA7 were grown in RPMI1640+5% foetal calf serum with selective agents used as described. HCA7 was grown in DMEM+10% foetal calf serum. DLD1 cells were cotransfected with 2 *μ*g of *λ*LIZ (prepared as above) and 500 ng pcDNA3.1 Hygro (Invitrogen) using Lipofectamine 2000 (Invitrogen) according to the manufacturer's protocol for 24 wells. After transfection, cells were seeded into 10 cm dishes and colonies were selected using 300 *μ*g ml^−1^ hygromycin B (Invitrogen). Colonies were screened for intact *λ*LIZ by PCR amplification using primer pairs spanning *lac*I and at the end of each arm of *λ*LIZ. Successful packaging of positive clones was checked using protocols described below. Cell lines with *λ*LIZ (T54c4, T54c10) were transfected with *Pvu*I-linearised *MBD4*^*tru*^.pcDNA3 expression plasmid or *Pvu*I-linearised empty vector, using Lipofectamine 2000 as described above, and colonies were selected using 600 *μ*g ml^−1^ Geneticin (Invitrogen).

### Methylation check of transfected *λ*LIZ

A portion of 1 *μ*g genomic DNA of cell lines was set up for overnight digestion with no enzyme, *Hpa*II or *Msp*I. Samples were then PCR amplified using primer pairs for ∼450 bp fragments of either *lacI* (2A and 13A, spanning two *Hpa*II/*Msp*I sites) or the CpG island of *MBD1* (59 and 60, spanning three *Hpa*II/*Msp*I sites). Where the CpG sites were methylated, *Hpa*II did not cut and PCR amplification could occur, whereas *Msp*I cuts independent of methylation. Controls for efficacy of digestion of DNA by *Hpa*II of unmethylated DNA were provided by the *MBD1* samples (*MBD1* being a constitutively expressed gene with unmethylated CpG island).

### Big Blue plaque assays and sequencing

Cell pellets were frozen at −70°C and then used for DNA extraction by the phenol–chloroform protocol as described in the Big Blue Instruction Manual (Stratagene). The *λ* shuttle vector containing the *lacI* target gene was recovered from genomic DNAs as described in the Big Blue Instruction Manual and packaged with *λ* packaging extract (Transpack, Stratagene). After initial dilution plating of packaging reactions, a density of 10 000 PFU per plate was targeted on 24.5 × 24.5 cm assay dishes, but each plating session was accompanied by a 1/40th dilution plating and all plaques counted for accurate assessment of the number of plaques screened. Blue mutant plaques were identified against a red background on a lightbox, and re-plated to verify as true mutants. Mutant frequency was calculated by dividing the number of blue plaques by the total number plated. Assays were performed for DNAs packaged from at least four independent pellets of cells per cell line, and usually between 250 000 and 400 000 PFU screened per packaged DNA. At least 30 well-separated mutant plaques per cell line were picked from plates for sequencing, starting from one corner of the plate and working inwards to the centre with each blue plaque encountered taken, so as to avoid any colour/mutation bias. Cored plaques were placed in SM buffer, from which an aliquot of buffer/phage suspension was taken and used as template in PCR amplification of the entire *lacI* gene (primers 1A and 8 as in the Stratagene manual). PCR products were then sequenced using [^33^P]dATP and Thermosequenase cycle sequencing kits (Amersham) from primers recommended in the Big Blue Instruction Manual (Stratagene), the products run on 6% acrylamide : urea gels and then exposed to autoradiographic film. To aid in visual screening for mutant bases, samples were run in batches of 10, with all G's together, all A's together and so on.

For analysis of mutation spectra, enough plaques were sequenced to identify about 50 independent mutations (defined as unique base changes at unique nucleotides, that is, disregarding multiple incidences in different plaques) for each cell line.

### Statistical analyses

Comparison of mutation frequencies was carried out by two-tailed unpaired *t*-test. Statistical comparisons of mutation spectra were carried out using the Monte Carlo approximation to Fisher's exact test of Adams and Skopek (1987), with 1700 iterations in a programme available on the web (Cariello *et al*, 1994) and the significance level set at 0.05. The test conducts pairwise comparisons using the 11 mutational classes shown in Figure 4B and Supplementary Table 2), including the subset of G:C to A:T mutations that occurred at CpG sites.

## RESULTS

### Preparation of recombinant proteins

**Figure 1:**
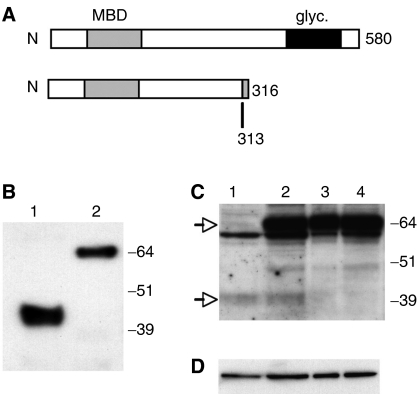
Expression of full-length (FL) and truncated (tru) MBD4. (**A**) Diagram of MBD4 proteins; the MBD4^tru^ sequence is the same as FL up to aa 313 after which there are three novel residues (KDH) owing to the frameshift. (**B**) Purified recombinant proteins 1: tru, 2: FL, detected by Western blot hybridisation with anti-His tag antibody. (**C**) MBD4 in nuclear fractions of colorectal carcinoma cell lines 1: HCA7 2: HCT116 3: T54c4 4: T72con1, detected by Western blot hybridisation with antibody H-300. Arrows indicate endogenous FL (upper) and tru (lower) MBD4. (**D**) Loading control Western blot using anti-lamin B.

We prepared recombinant His-tagged wild-type/full-length (FL-MBD4) and truncated (MBD4^tru^) human MBD4 proteins for use in glycosylase assays. MBD4^tru^ comprises wild-type protein sequence from aa 1–313 with three further novel residues 314–316, and includes the MBD (aa 80–146) with 167 aa of intervening sequence (see Figure 1A). For MBD4^tru^, we amplified PCR exons 1, 2, and part of 3 of the gene from cDNA of HCT116 cells that are heterozygous (A_10_/A_9_) and express both alleles (Bader *et al*, 1999). Following transformation, bacterial colonies were screened by PCR and cycle sequencing for the presence of A_9_ *vs* A_10_ allele, after which the entire cDNA and flanking plasmid regions of a candidate colony were sequenced to rule out mutations that may have arisen during the cloning procedure. Recombinant proteins were then expressed and purified. To confirm the apparent molecular weights of the two purified recombinant proteins as seen on a Western immunoblot probed with anti-His tag antibody (Figure 1B), we used linearised plasmids to express protein from plasmid T7 promoters in rabbit reticulocyte lysates and then carried out SDS–PAGE (data not shown). Plasmids used contained MBD4 cDNAs cloned in both the prokaryotic expression vector and the eukaryotic expression vector transfected in experiments described later. In each case, full-length MBD4 ran at an apparent molecular weight of about 66 kDa and truncated MBD4 at about 43 kDa. The value for full-length protein is as expected from its theoretical molecular weight, but is about 5 kDa larger for truncated protein. Furthermore, Western analysis of nuclear proteins of HCT116 (heterozygous wt/mut) and HCA7 (homo- or hemi-zygous mutant) colorectal carcinoma cell lines with H-300 antibody detected both forms of MBD4 (Figure 1C). Only full-length MBD4 was seen in T54c4 (DLD1 cells with endogenous wt*MBD4* and transfected only with *λ*LIZ Big Blue shuttle vector) and T72con1 (T54c4 transfected with empty pcDNA vector). Truncated MBD4 was expressed in *MBD4*-mutant tumour cells albeit at an apparently lower level. Barring any unknown post-translational modifications that might reduce the affinity of H-300-binding to MBD4^tru^, we predict that the lower level of MBD4^tru^ is due to nonsense-mediated decay of mRNA, typical of such mutations (El-Bchiri *et al*, 2005). Figure 1D shows the Western blot hybridisation for loading control nuclear protein lamin B.

### Dominant negative inhibition in glycosylase assays

**Figure 2:**
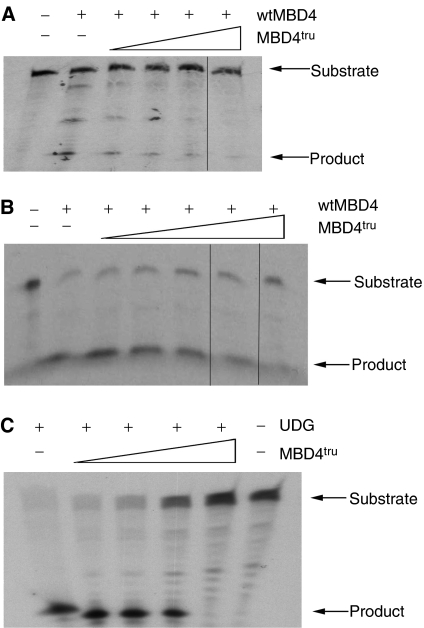
Dominant negative inhibition of glycosylase activities in cell-free glycosylase assays. (**A**) Oligo substrate BH:BHrev containing T.G mismatch, and FL-MBD4 competed with MBD4^tru^. (**B**) Oligo substrate MM2:MM3revU containing U.G mismatch, and FL-MBD4 competed with MBD4^tru^. (**C**) Oligo substrate BH:BHrevU containing U.G mismatch, and UDG competed with MBD4^tru^. In each case, ‘+’ indicates presence and ‘−’ absence of protein, and the wedge indicates relative concentration of MBD4^tru^ in roughly two-fold increments from 1 to 10 (**A**) or from 1 to 20 (**B**, **C**). Vertical lines indicate where lanes have been juxtaposed owing to removal of an intervening, non-two-fold incremental lane.

Cell-free assays with the purified recombinant proteins showed that MBD4^tru^ could inhibit T·G glycosylase activity of full-length MBD4 (Figure 2A). In this and the following assays, the amount of cut oligo product generated by the activity of the wild-type glycosylase was seen to decrease in the presence of increasing amounts of MBD4^tru^, with an increase in the uncut oligo substrate above. As MBD4 has been shown to be an efficient U·G glycosylase, we tested the ability of MBD4^tru^ to inhibit this function, and found that inhibition can also be seen (Figure 2B). Our assays also showed that MBD4^tru^ could competitively inhibit U·G glycosylase activity of another DNA repair enzyme that recognises U·G mismatches, Uracil DNA glycosylase (UDG) (Figure 2C).

### Big Blue cell lines

**Figure 3:**
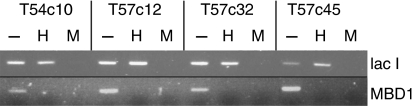
Methylation of transfected *λ*LIZ shuttle vector. Genomic DNA of cell lines was digested with *Hpa*II (H), *Msp*I (M) or treated with no enzyme (−), and then PCR amplified using primer pairs to amplify a CpG-rich part of (**A**) transfected *λ*LIZ or (**B**) endogenous MBD1 gene. Cell lines shown are T54c10 (clone of DLD1 +*λ*LIZ) and T57c12, c32, 45 (all clones of T54c10 +MBD4^tru^).

The Big Blue *λ*LIZ reporter shuttle vector containing part of the *lacI-lacZ* operon (Stratagene) was transfected into DLD1 cells that are wild-type for *MBD4*. Although cellular methylation of transfected LIZ was expected to occur as such foreign, silent DNA usually undergoes this modification (as was the case Big Blue mice; You *et al*, 1998), we wished to guarantee methylation of *λ*LIZ CpG sites at the outset and so pretreated *λ*LIZ with SssI methylase before transfection. Because *λ*LIZ does not have a selectable marker, it was cotransfected with pcDNA3 Hygro3.1 and clones were selected with hygromycin B. Clones were screened by PCR for presence of *λ*LIZ and tested for packaging ability, before being used for MF assays and further transfected with *MBD4*^*tru*^ cDNA. Two clones, T54c10 and T54c4 were generated, and they that carried intact, concatenated *λ*LIZ and packaged efficiently. Methylation of the integrated *λ*LIZ was confirmed by PCR amplification of DNA digested with *Hpa*II or *Msp*II, spanning a region of *lac*I containing two *Hpa*II*/Msp*I sites (Figure 3). Efficacy of *Hpa*II digestion was confirmed by PCR amplification of part of the CpG island of an expressed, unmethylated gene (*MBD1)* from the same digested DNA samples (Figure 3). The endogenous MBD4 genes of both T54c4 and T54c10 were also PCR amplified and sequenced to confirm their polyA tract wild-type status. These two clones were then transfected with *MBD4*^*tru*^ cDNA expression plasmid or empty vector alone, and geneticin-resistant clones were isolated. *MBD4*^*tru*^ cDNA-containing clones were confirmed by PCR, and expression of the exogenous gene was verified by Western blot hybridisation (Supplementary Figure 1).

### MBD4^tru^ increases mutation frequency

**Figure 4:**
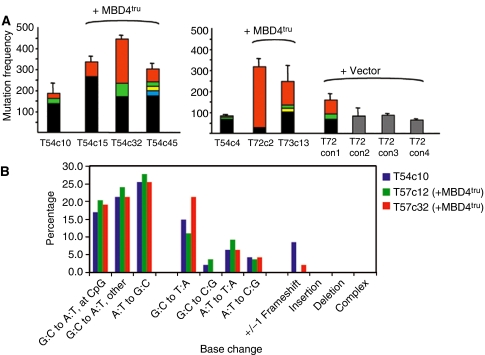
Big Blue mutation frequencies and spectra of cell lines ±MBD4^tru^. (**A**) The left panel shows T54c10 and transfected (+MBD4^tru^) derivatives as indicated, whereas the right panel shows T54c4 and transfected (+MBD4^tru^) derivatives. The *Y*-axis shows the mutation frequency of each cell line, × 10^−5^ with bars showing the standard deviation. Within each column the colours represent subsets of mutations, according to the proportions of plaques sequenced: black – independent mutations (frequency ⩽6% those sequenced); grey – not sequenced; other colours – jackpot mutations (frequency >6%) (NB: the same colour in different columns does not necessarily indicate the same mutation base change). (**B**) Mutation spectra of T54c10 and two clones +MBD4^tru^. The heights of columns indicate the percentage incidence of each type of base change.

We next determined the mutation frequency (MF) of a human MSI colon cancer cell line, DLD1, with/without MBD4^tru^we found that the total MF increased in all clones transfected with MBD4^tru^. The background spontaneous Big Blue mutation frequencies of the two parental clones of DLD1 were 79 × 10^−5^ (T54c4) and 178 × 10^−5^ (T54c10) (Table 1). Expression of exogenous MBD4^tru^ increased the MF of T54c4 to 292–310 × 10^−5^ (two clones tested), and for T54c10 to 306–440 × 10^−5^ (three clones tested) (all *P*-values <0.05) (Table 1 and Supplementary Table 1). This is an increase in total MF of about 2–4 fold above that for parental cells and indicates increased genomic instability beyond that due to pre-existing MSI. Furthermore, sequencing of mutant plaques revealed that a significant proportion of blues for each clone tested carried one of several identical nucleotide changes (a ‘jackpot’ (Heddle 1999) mutation, that is, the same base change at the same base-pair location and defined in our experiments as accounting for >6%, or seen in two or more of 30 sequenced plaque samples) as shown in Table 2 and Figure 4A. These identical changes represent more likely a provenance as offspring from the same parental cell that had suffered a mutation early in the expansion of the clone after transfection, rather than independent *de novo* mutations. Normally in Big Blue experiments using drug-treated animals, jackpot mutations are disregarded as confounding data resulting from clonal expansion in a subset of cells created during tissue development *in vivo*. However, given the structure of the *in vitro* experiments described here involving sequential transfection of individual cells with the applied stress factor (expressed mutant gene, rather than drug applied to a whole mixed cell population), the jackpots enable us to evaluate further the increased MF. Allowing for chance occurrence of a mutation at any given time followed by clonal expansion, the data can be interpreted as follows: the greater is the jackpot effect (number of jackpots and/or proportion of the MF), the greater is the underlying genomic instability. Furthermore, the relative proportion of MF accounted for by any one jackpot gives an indication of how early during clonal expansion after transfection that jackpot occurred. The extreme example of this is the T72c2 cell line where, of the 30 plaques sequenced, 28 carried the same mutation, suggesting an event that occurred very early after transfection and selection.

As expected, three of the four control clones transfected with empty expression vector (i.e. no applied stress factor) displayed a range of MF, but roughly equivalent to the parental clone T54c4. One control, T72con1, did have an MF almost double that of its parent and this was found to be due to jackpots. Presumably in this particular instance, these jackpots occurred as a result of the innate MSI of the T54c4 parent, but by chance earlier during clonal expansion than for its parental or sibling control clones. The results therefore confirm that the parental DLD1 MSI cell line has an MF that is itself higher than that for normal, genomically stable somatic cells. It also highlights that the sensitivity of this assay system to detect small changes in mutation frequency is limited. In addition, our data demonstrate that genomic instability, as evinced by overall MF and the proportion of MF owing to an increased number of jackpots, increases further in the presence of MBD4^tru^.

### MBD4^tru^ affects a wide mutation spectrum

The mutation spectra of three cell lines (parental T54c10 and two +MBD4^tru^ clones T57c12 and T57c32) were determined by sequencing about 50 independent mutations. These spectra showed that the mutations occurring in the presence of MBD4^tru^ spanned the spectrum of possible changes with no bias towards G:C to A:T transitions at CpG sites and no significant difference from the parental cell line containing only wild-type MBD4 (T54c10 *vs* T57c12 *P*=0.48, T54c10 *vs* T57c32 *P*=0.52) (Figure 4B and Supplementary Table 2). In addition, the base changes found in the jackpots included examples of several types of base change (Table 2). This is in contrast to the shift of mutation spectra in the absence of Mbd4 in the mouse model, where the predominant change seen is an increase in C to T transitions at methyl-CpG sites.

## DISCUSSION

The role of MBD4 was initially thought to be limited to the detection and repair of T·G mismatches that occur at methyl-CpG dinucleotides as a result of deamination of the methyl-C. This was demonstrated by the behaviour of recombinant protein in cell-free gel-shift and glycosylase systems. Some other base-pair mismatches affecting CpG dinucleotides were also shown to be lower affinity substrates for MBD4 activity (Petronzelli *et al*, 2000a, 2000b) and as time has progressed more substrates have been documented, at least *in vitro* (Liu *et al*, 2002; Yoon *et al*, 2003). The role of MBD4 as a methyl-CpG-focused DNA mismatch glycosylase was subsequently supported by increases in mutation frequency at CpG sites in mouse models lacking Mbd4 (Millar *et al*, 2002; Wong *et al*, 2002). However, we and others have found an association of truncating mutations of *MBD4* in human MSI cancers, especially colon, and in most cases these mutations occur in the presence of a remaining intact *MBD4* allele (Bader *et al*, 1999, 2000; Riccio *et al*, 1999), that is, the null genotype is not seen naturally. As the MBD of MBD4 was reported to be able to bind methylated oligos with a T·G mismatch, we have hypothesised that a dominant negative activity could be present in cells heterozygous for an MBD4^tru^ mutation that has occurred as a result of prior MMR gene abnormality. In the absence of any known dimerisation of MBD4 molecules for activity, we envision the inhibition to occur via steric recognition and competition for binding sites. In this study, we have shown that MBD4^tru^ can indeed inhibit glycosylase activity of wtMBD4 and at least one other glycosylase (UDG) in cell-free assays.

We went on to determine the mutation frequencies of living cells using the *λ*LIZ shuttle vector as an indicator. This is the first time that the Big Blue system has been reported using human cells so there are no prior reports of MF of the *lacI* gene in human cells or tissues for comparison. However, the values of the parental clones T54c10 and T54c4 are between 16 × and 90 × the spontaneous MF of *λ*LIZ in somatic tissues of untreated Big Blue mice (*lacI* reporter system) and a similar *lacZ* transgenic mouse reporter system, ranging from 2 to 5 × 10^−5^ in several reports (Morrison and Ashby, 1994). The high MF of these clones of DLD1 is due to the defect in the *MSH6* MMR gene of this cell line, and similarly, high MF of DLD1 or other MSI colon cancer cells compared with normal diploid fibroblasts has been reported elsewhere using the *HPRT* or *ouabain*^*R*^ endogenously expressed gene systems (Bhattacharyya *et al*, 1994, 1995). We found that expression of MBD4^tru^ roughly doubles mutation frequency in cells even on the background of MMR defect, on agreement with the dominant negative hypothesis. The increase in genomic instability was indicated not only by an increase in overall MF, but also the relative effect of jackpot mutations. The latter observation was also a novel interpretation of Big Blue data, useful in this instance of cell culture rather than the usual animal experiments.

The first report of substrates of MBD4 showed preference for T·G products of methyl-C deamination within methyl-CpG sites (Hendrich *et al*, 1999). Later reports demonstrated a larger set of *in vitro* CpG mismatches for MBD4 recognition, such as U·G (Petronzelli *et al*, 2000a, 2000b), 5-fluorouracil (5-FU)·G (Petronzelli *et al*, 2000b), ethenecytosine (*ε*C)·G (Petronzelli *et al*, 2000a), 5-formyluracil (5-FoU)·G (Liu *et al*, 2002), T·O^6^-methyl-guanine (O^6^-meG) (Cortellino *et al*, 2003) and thymine glycol (Tg)·G (Yoon *et al*, 2003). Total absence of MBD4 in mice caused an increase in MF especially at CpG sites (Millar *et al*, 2002; Wong *et al*, 2002). This was as predicted from the originally reported range of substrates and by the fact that the other main T·G mismatch enzyme, Thymine DNA glycosylase (TDG), does not have an MBD and so does not focus on methyl-CpG sites. Repair of T·G and U·G mismatches at non-methylated CpG sites, an Mbd4 function lost in Mbd4-null cells, would be covered by redundant glycosylases TDG and UDG. We had predicted that dominant negative action of MBD4^tru^ would likewise increase C to T transitions, especially at methyl-CpG sites in human cells, owing to inhibition of wtMBD4 with some gain-of-function inhibition of TDG and UDG. Our observation that the mutation spectrum in the presence of MBD4^tru^ does not specifically show an increase of C to T transitions at methyl-CpG sites, but instead increases mutation frequency across the entire spectrum, implies that MBD4^tru^ not only inhibits wtMBD4 activity at methyl-CpG sites, but also activities of the other glycosylases at least, and perhaps also other DNA repair pathways. This conclusion is consistent with the data implicating MBD4 in a wider role of DNA damage substrate recognition and repair leading to apoptosis in Mbd4-null mice (Sansom *et al*, 2003; Screaton *et al*, 2003) treated with for example the methylating agent temozolamide and the crosslinking chemical cisplatin. The important difference between the mouse studies and this is that the effects presented here occurred owing to expression of a naturally occurring truncation mutant of MBD4 found in authentic human tumours. The agreed sequence of events in human tumours involves a mutational event that leads to an MMR gene abnormality thus causing the MSI phenotype that then by chance hits an *MBD4* allele. The dominant negative activities of MBD4^tru^ in our experiments suggest that wtMBD4 is involved directly in more than just methyl-CpG repair or that MBD4^tru^ is able to interfere nonspecifically in repair systems, or both. The former is consistent with the data from Mbd4 knockout experiments. The increase in MF seen here following relatively short-term culture with a high level of expression of truncated MBD4 is demonstration in principle of the dominant negative effect of this mutant protein. In the physiological situation with expression of endogenous truncated MBD4 at a relatively lower level because of NMD, the increase in MF is also expected to be less. In this way, NMD has an important role in protecting cells from damaging activities of frameshifting mutations. However, in view of the stochastic nature of mutations arising as a result of perturbed DNA repair, over the extended period of time of 5–20 years that it takes to get malignant colon tumours, even in patients with MSI owing to HNPCC, it is feasible for even the low level of MBD4^tru^ to compound the genomic instability owing to MMR defects. Such dominant negative interference with a tumour cell's biology may also have relevance to therapeutic intervention. We are in the process of using the same cell lines to test for changes in sensitivity to DNA-damaging agents with respect to signaling to apoptosis, and it is possible that cells carrying MBD4^tru^ mutations will also be more tolerant of the types of damage tested in Mbd4-null systems. It will also be important to study the effects of MBD4^tru^ at more physiological levels, for example, by creating a mouse knock-in model whereby a truncated Mbd4 allele is expressed under the control of the endogenous promoter.

## Figures and Tables

**Table 1:** Big Blue mutation frequencies in cell lines, summary

**Cell line**	**MBD4^tru^**	**mean MF × 10^−5^**	**s.d.**	***P***	**Relative MF increase^a^**
T54c10	−	178	47		
T57c12	+	327	28	2.27 × 10^−4^	1.8
T57c32	+	440	19	8.64 × 10^−6^	2.5
T57c45	+	306	28	3.38 × 10^−3^	1.7

T54c4	−	79	6		
T72c2	+	310	37	8.27 × 10^−8^	3.9
T73c13	+	242	131	1.34 × 10^−3^	3.1
T72con1	−	157	30	4.83 × 10^−4^	2.0
T72con2	−	81	41	0.64	1.0
T72con3	−	83	9	0.45	1.0
T72con4	−	60	5	7.23 × 10^−4^	0.8

a ‘relative increase’ compared with –MBD4^tru^ parental cell line T54c10 or T54c4

**Table 2:** Jackpot mutations

**Cell line**	**MBD4^tru^**	**No. plaques sequenced**	**No. jackpots**	**Total % of MF owing to jackpots**	**Base changes**
T54c10		76	2	25	A to G, T to G
T57c12	+	97	1	20	T to A
T57c32	+	124	2	61	A to G, T to C
T57c45	+	30	4	40	T to C, A to G, G to A (in CpG), C to T

T54c4		30	2	17	C to T, T to C
T72c2	+	30	1	93	G to A
T73c13	+	30	3	60	C to T, G to A, A to T
T72con1	+	30	2	57	T to C
