# Supplementary material for: A human cancer-associated truncation of MBD4 causes dominant negative impairment of DNA repair in colon cancer cells
Source: Br J Cancer. 2007 Feb 6;96(4):660–6. doi: 10.1038/sj.bjc.6603592 (PMC2360052; doi:10.1038/sj.bjc.6603592)
Supplement: Supplementary Table 1 [file 6603592x2.doc]

Supplementary Table 1 - Mutation frequency data details

| Parental |  | assay | no.pfu | number | Mutation | s.d. | relative |  |
| --- | --- | --- | --- | --- | --- | --- | --- | --- |
| cell line | clone | no. | screened | mutants | Freq. |  | MF | p = |
|  |  |  |  |  | (MF) |  |  |  |
| T54c10 |  | 1 | 327180 | 465 | 142 |  |  |  |
|  |  | 2 | 296100 | 400 | 135 |  |  |  |
|  |  | 3 | 297000 | 598 | 201 |  |  |  |
|  |  | 4 | 300900 | 700 | 233 |  |  |  |
|  |  | **MEAN** |  |  | **178** | **47** | **1** |  |
|  |  |  |  |  |  |  |  |  |
|  | T57c12 | 5 | 315740 | 1039 | 329 |  |  |  |
|  | +MBD4tru | 6 | 234680 | 776 | 331 |  |  |  |
|  |  | 7 | 256380 | 890 | 347 |  |  |  |
|  |  | 8 | 287980 | 983 | 341 |  |  |  |
|  |  | 9 | 245200 | 835 | 340 |  |  |  |
|  |  | SBB8 | 338320 | 918 | 271 |  |  |  |
|  |  | **MEAN** |  |  | **327** | **28** | **1.8** | **2.27E-04** |
|  |  |  |  |  |  |  |  |  |
|  | T57c32 | 10 | 319160 | 1482 | 464 |  |  |  |
|  | +MBD4tru | 11 | 318440 | 1414 | 444 |  |  |  |
|  |  | 12 | 299820 | 1246 | 415 |  |  |  |
|  |  | 13 | 344720 | 1477 | 428 |  |  |  |
|  |  | 14 | 331872 | 1492 | 449 |  |  |  |
|  |  | **MEAN** |  |  | **440** | **19** | **2.5** | **8.64E-06** |
|  |  |  |  |  |  |  |  |  |
|  | T57c45 | 15 | 169040 | 465 | 275 |  |  |  |
|  | +MBD4tru | 16 | 264780 | 843 | 318 |  |  |  |
|  |  | 17 | 301340 | 882 | 293 |  |  |  |
|  |  | 18 | 254060 | 859 | 338 |  |  |  |
|  |  | **MEAN** |  |  | **306** | **28** | **1.7** | **3.38E-03** |
|  |  |  |  |  |  |  |  |  |
| T54c4 |  | 19 | 278020 | 239 | 86 |  |  |  |
|  |  | 20 | 207520 | 161 | 78 |  |  |  |
|  |  | 21 | 345740 | 286 | 83 |  |  |  |
|  |  | 22 | 261580 | 200 | 76 |  |  |  |
|  |  | 23 | 334200 | 234 | 70 |  |  |  |
|  |  | **MEAN** |  |  | **79** | **6** | **1** |  |
|  |  |  |  |  |  |  |  |  |
|  | T72c2 | 24 | 310120 | 994 | 320 |  |  |  |
|  | +MBD4tru | 25 | 306860 | 691 | 313 |  |  |  |
|  |  | 26 | 316080 | 1079 | 341 |  |  |  |
|  |  | 27 | 417780 | 1383 | 331 |  |  |  |
|  |  | 28 | 303040 | 1053 | 347 |  |  |  |
|  |  | 29 | 334992 | 888 | 265 |  |  |  |
|  |  | 30 | 313320 | 793 | 253 |  |  |  |
|  |  | **MEAN** |  |  | **310** | **37** | **3.9** | **8.27E-08** |
|  |  |  |  |  |  |  |  |  |
|  | T73c13 | 31 | 276600 | 847 | 306 |  |  |  |
|  | +MBD4tru | 32 | 314120 | 364 | 116 |  |  |  |
|  |  | 33 | 290540 | 788 | 271 |  |  |  |
|  |  | 34 | 293480 | 846 | 288 |  |  |  |
|  |  | 35 | 304640 | 705 | 231 |  |  |  |
|  |  | **MEAN** |  |  | **242** | **76** | **3.1** | **1.34E-03** |
|  |  |  |  |  |  |  |  |  |
|  | T72con1 | 36 | 324800 | 532 | 163 |  |  |  |
|  | +vector | 37 | 302080 | 507 | 168 |  |  |  |
|  |  | 38 | 297720 | 551 | 185 |  |  |  |
|  |  | 39 | 286040 | 466 | 163 |  |  |  |
|  |  | 40 | 287640 | 302 | 105 |  |  |  |
|  |  | **MEAN** |  |  | **157** | **30** | **2** | **4.83E-04** |
|  |  |  |  |  |  |  |  |  |
|  | T72con2 | 41 | 397520 | 319 | 80 |  |  |  |
|  | +vector | 42 | 296160 | 181 | 61 |  |  |  |
|  |  | 43 | 337360 | 463 | 137 |  |  |  |
|  |  | 44 | 393040 | 193 | 49 |  |  |  |
|  |  | 45 | 382440 | 200 | 52 |  |  |  |
|  |  | 46 | 308700 | 143 | 46 |  |  |  |
|  |  | **MEAN** |  |  | **81** | **41** | **1** | **0.64** |
|  |  |  |  |  |  |  |  |  |
|  | T72con3 | 47 | 294540 | 218 | 74 |  |  |  |
|  | +vector | 48 | 325980 | 320 | 98 |  |  |  |
|  |  | 49 | 330720 | 252 | 76 |  |  |  |
|  |  | 50 | 374340 | 308 | 82 |  |  |  |
|  |  | 51 | 382320 | 318 | 83 |  |  |  |
|  |  | **MEAN** |  |  | **83** | **9** | **1** | **0.45** |
|  |  |  |  |  |  |  |  |  |
|  | T72con4 | 52 | 336280 | 192 | 57 |  |  |  |
|  | +vector | 53 | 429520 | 257 | 60 |  |  |  |
|  |  | 54 | 218880 | 120 | 55 |  |  |  |
|  |  | 55 | 245720 | 167 | 68 |  |  |  |
|  |  | 56 | 272880 | 158 | 58 |  |  |  |
|  |  | **MEAN** |  |  | **60** | **5** | **0.8** | **7.23E-04** |
