# Supplementary material for: A human cancer-associated truncation of MBD4 causes dominant negative impairment of DNA repair in colon cancer cells
Source: Br J Cancer. 2007 Feb 6;96(4):660–6. doi: 10.1038/sj.bjc.6603592 (PMC2360052; doi:10.1038/sj.bjc.6603592)
Supplement: Supplementary Table 2 [file 6603592x3.doc]

| mutation type | T54c10 | | T57c12 | | T57c32 | |
| --- | --- | --- | --- | --- | --- | --- |
|  | no. | % | no. | % | no. | % |
| G:C to A:T, at CpG | 8 | 17.0 | 11 | 20.4 | 9 | 19.1 |
| G:C to A:T, other | 10 | 21.3 | 13 | 24.1 | 10 | 21.3 |
| A:T to G:C | 12 | 25.5 | 15 | 27.8 | 12 | 25.5 |
|  |  |  |  |  |  |  |
| G:C to T:A | 7 | 14.9 | 6 | 11.1 | 10 | 21.3 |
| G:C to C:G | 1 | 2.1 | 2 | 3.7 | 0 | 0.0 |
| A:T to T:A | 3 | 6.4 | 5 | 9.3 | 3 | 6.4 |
| A:T to C:G | 2 | 4.3 | 2 | 3.7 | 2 | 4.3 |
|  |  |  |  |  |  |  |
| +/-1 frameshift | 4 | 8.5 | 0 | 0.0 | 1 | 2.1 |
| insertion | 0 | 0.0 | 0 | 0.0 | 0 | 0.0 |
| deletion | 0 | 0.0 | 0 | 0.0 | 0 | 0.0 |
| complex | 0 | 0.0 | 0 | 0.0 | 0 | 0.0 |
|  |  |  |  |  |  |  |
| Total | 47 | 100.0 | 54 | 100.1 | 47 | 100.0 |

Supplementary Table 2. Mutation spectra details
