# Supplementary material for: A human cancer-associated truncation of MBD4 causes dominant negative impairment of DNA repair in colon cancer cells
Source: Br J Cancer. 2007 Feb 6;96(4):660–6. doi: 10.1038/sj.bjc.6603592 (PMC2360052; doi:10.1038/sj.bjc.6603592)
Supplement: Supplementary Table 3 [file 6603592x4.doc]

Supplementary table 3 – oligo sequences

Cloning of MBD4tru cDNA for expression of recombinant protein

4NcoMet ACG TCC ATG GGC ACG ACT GGG CT

MBD4.12Bam ACG TGG ATC CAG AAC AAA AAT TTG ATC CTG AAC TC

Glycosylase assays

(M= methyl-C)

BH TCA GAT TCG CGC MGG CTG CGA TAA GCT GMG CGG ATC CMG GGA ATT CAG CT

BHrev (Y= T) AGC TGA ATT CCY GGG ATC CGY GCA GCT TAT CGC AGC YGG CGC GAA TCT GA

BHrevU (Y= U) AGC TGA ATT CCY GGG ATC CGY GCA GCT TAT CGC AGC YGG CGC GAA TCT GA

MM2 TCA GAT TCG CGC MGG CTG CGA TAA GCT

MM3revU AGC TTA TCG CTG CCU GCG CGA ATC TGA

Cloning of MBD4tru cDNA for expression in cell lines

MBD4.1a TGG GCT CGT TGC TGC AG

MBD4.14 CTT TCC TTT CCA CAA CTT CTA CT

Screening of clones for intact λLIZ

Left 1 ACT CCG CTG AAG TGG TGG A

Left 2 CAC GTC TCA CCG AGC GTG

Right 1 GCG TGG AAA GAT TTG TGT AGT GT

Right 2 GTC GGA GAC TAA CGT CAG AA

Methylation specific PCR

lacI.2A CCC GAC ACC ATC GAA TG

lacI.13A CCA GCG GAT AGT TAA TGA TCA

MBD1.59 GAG CCT CCC TAT TAA GGG CA

MBD1.60 CTG TCA GAG TTC AGA GCT GCA
